# Policy makers’ perceptions of the high burden of heart disease in rural Australia: Implications for the implementation of evidence-based rural health policy

**DOI:** 10.1371/journal.pone.0215358

**Published:** 2019-04-16

**Authors:** Laura Alston, Melanie Nichols, Steven Allender

**Affiliations:** Global Obesity Centre (GLOBE), Faculty of Health, Deakin University, Geelong, Australia; University of Bristol, UNITED KINGDOM

## Abstract

**Background:**

Rural Australian populations experience an increased burden of ischaemic heart disease (IHD) compared to their metropolitan counterparts, similar to other developed countries, globally. Policy and other efforts need to address and acknowledge these differences in order to reduce inequalities in health burden. This paper examines rural health policy makers’ perceptions and use of evidence in efforts to reduce the burden of IHD in rural areas.

**Methods:**

Policy makers and government advisors (n = 21) who worked with, or advised on, rural health policy at local, state and federal government levels, with specific focus on the state of Victoria (n = 9) were identified from publicly available documents and subsequent snowball sample. Semi-structured qualitative interviews were conducted in regards to the use of evidence in policy to prevent IHD and thematic analysis undertaken applying two theoretical perspectives: context-based evidence-based policy making and the conceptual framework for understanding rural and remote health.

**Results:**

The rural context, particularly low resourcing, was seen as limiting potential for evidence based policy at local government (LG) level. Lower levels of political pressure and education were seen as constraints to evidence-based policy in rural communities. Participants described the potential for policy to have a greater impact on reducing heart disease in rural areas though they felt under-resourced and out of touch with the scientific evidence. Scientific studies were less valued than local anecdote to prioritise specific policy. At all levels (local, state and federal) low self-efficacy in interpreting evidence and perceived lack of relevance inhibited development of evidence informed policy.

**Conclusion:**

The rural context constrains the use of scientific evidence in policy making for the prevention of heart disease in rural areas in Australia with multiple factors influencing the capacity for evidenced based health policy. This is similar to findings at the international scale and is for consideration across other developed countries that experience inequalities in IHD disease burden between rural and urban populations.

## Introduction

Cardiovascular diseases (CVD), including ischaemic heart disease (IHD) are the leading causes of death in Australia[1,2]. Rural and remote dwelling Australians experience a higher and disproportionate burden of these diseases when compared to their metropolitan based counterparts[3,4].

A large inequality in CVD burden persists between rural and metropolitan Australia, and in 2015, rural Australians were reported to be between 1.15 and 1.3 times more likely to die from CVD than their metropolitan counterparts [5]. A recent study using macro-simulation modelling techniques (the PRIME model) suggested that almost 40% of the gap in deaths between rural and metropolitan areas would be prevented if modifiable risk factor profiles among rural populations mirrored the those in metropolitan areas [6]. Rural populations with CVD worldwide have been shown to have a lower prevalence of healthy lifestyle attributes than their urban counterparts and this presents as an ongoing challenge for public health policy and action [7].

The persistent inequity in disease burden between rural and metropolitan areas is in part due to current and historical public health policies serving metropolitan populations more effectively than rural Australians [6,8–11]. Inequalities in CVD burden across rural areas in low, middle and high-income countries have been documented worldwide, with evidence to show that systemic change and action is needed in rural communities across the primary, secondary and tertiary CVD prevention contexts [7,12,13].

The use of evidence in policy has been encouraged in light of ‘evidence based practice’ (EBP) which forms the basis and justification of strategies for clinical health interventions in modern medicine. However, this practice is much less common in the practice of health policy and decision making at the population level [14,15]. In the clinical context, evidence-based guidelines for treatment of heart disease, such as acute coronary syndrome, specifically consider geographical challenges and requirements in the rural context and have been developed as a result of evidence of the differences between rural and urban Australian populations [16]. A priori it seems reasonable that health policy in rural Australia should use a more evidence led approach [17] in creating health policy addressing non-communicable disease (NCD) risk that is specific to the rural context [14,15,18]. In reality implementation of evidence based policy appears to have been hampered by competing agendas, shifting ‘policy windows’, differing government priorities [19,20] and electoral promises, political pressures, resources and the individual values [14,15,17,18]. As inequalities persist, there is very little research on the use of evidence within rural health policy.

There are many external factors (such as the rural context) that ultimately influence how and if evidence is used to justify health policy decisions[15]. To understand the use of evidence in rural health policy, two pertinent frameworks are useful, being *The Conceptual Framework of Context-Based Evidence-based Decision Making* by Dobrow et al (2004) [15] combined with *The Conceptual Framework for Understanding Rural and Remote Health* by Bourke et al (2012)[21]. The *Conceptual Framework of Context-Based Evidence-Based Decision Making* has been used to guide qualitative analysis to better understand evidence use in the development of breast cancer screening[22] and colorectal cancer screening policy[15] in varying health care settings. To understand the influence of the rural context in the process of using the scientific evidence to drive health policy, the *Conceptual Framework for Understanding Rural and Remote Health*[23] provides a lens specific to the unique social, cultural and spatial conditions observed in rural Australia. This framework has previously been used to understand how the rural context affects policy planning for primary health care services in rural areas and presents a way of defining the influence of the rural health system on health outcomes in Australia [23]. Bringing these two frameworks together may provide additional insight into the use (or not) of evidence about inequalities in the policy platforms seeking to improve rural IHD rates. In this study, for ease of description the term ‘rural’ refers to any location outside of a major city in Australia[24], the term ‘policy’ [25] is any intentional government policy aimed at reducing the IHD burden in rural areas, and finally, the term ‘evidence’ refers to the research or published scientific evidence or data[26].

The aims of the study were to:

1. Describe the perceptions of evidence showing the increased IHD disease burden, among rural health policy makers and advocates in Victoria and compare these to views among their state and federal counterparts, and
2. Identify the extent to which there is adoption of evidence in Australian health policy in the unique rural context, and facilitators and barriers to adoption and implementation.
3. Consider the influence of the rural context over the use of scientific evidence to drive policy in rural Australia through the lens of two published conceptual frameworks [15,21].

## Methods

This research was conducted with assumptions informed by a post-positivist stance (26). Post-positivism argues that the truth can be uncovered and described, but never completely understood (26). Semi-structured qualitative interviews (n = 21) were conducted with policy makers and government advisors, working with or advising on rural health policy at local, state and federal levels, with specific focus at local level in Victoria (n = 9). Perspectives of those working at local government (LG) level in the state of Victoria were compared with Victorian state government and federal perspectives on the issue of IHD and rural health policy. See S1 File for an outline of the interview questions. Interviews were conducted to the point of data saturation whereby no new themes were emerging from the data, and repetition was emerging between participant responses. In qualitative research methods, data saturation indicates adequate participant sampling has occurred in the context of the research question[27]. Ethics approval was received from the Deakin University Human Ethics Advisory group within the faculty of Health reference number HEAG-H 91_2016.

### Recruitment

Participants were identified purposefully and via a snowball sampling method. To be eligible, participants had to have been working with health policy in a rural area for at least 1 year at either local or state level in Victoria, or at a federal level. Roles of participants included health policy makers, politicians, academics and leaders of relevant health advocacy organisations (e.g. NGOs) who worked directly with government policy makers (see Table 1). The state of Victoria was chosen as the state of focus, as all local governments in Victoria are required by legislation to have a strategic health and wellbeing plans under each council, and this is guided by the Public health and Wellbeing Act (2008)[28]. These plans formed the basis for enquiry of rural health policy at local level. States such as Western Australia do not have such requirements at local government level and therefore were not able to be investigated here. Recruitment was closed when the data reached thematic saturation, whereby no new themes emerged from the data[29], after 21 interviews. There were only a small number (n = 3) of interviews with state level participants as these participants had closely aligned views with National level participants, and participants generally had experience consulting to national level as needed, within their roles.

**Table 1:** Details of participants recruited for interviews, including the level of government they predominantly work within and a non-identifiable summary of their current/previous roles.

Role	Government level	Number of participants
**Policy developer**	Local (Victoria)	9
**Policy advisor**	State (Victoria)	2
**Member of Parliament**	State (Victoria)	1
**Advocate/ Policy advisor/ Senior academic**	National	9

### Interviews

Interviews were conducted either in person at the participant’s workplace, or via telephone by the lead researcher (LA) and audio-recorded. An interview schedule was developed using open ended questions around the following domains:

1. Perceptions of the increased burden of CVD/IHD in rural areas and barriers to effective policy actions
2. Priorities for health in rural areas
3. The use and perceptions of scientific evidence in the rural health policy making process
4. Barriers to using the scientific evidence in the policy and priority setting process

Interview times ranged from 25 minutes to 1 hour. All interviews were recorded and transcribed, and the interviewer also took notes throughout the interview process on additional observations.

### Analysis

Interviews were transcribed and checked for accuracy by LA. Transcripts were thematically analysed using a theoretical thematic analysis technique [27] which incorporates the use of a framework derived from the literature when defining themes within the data.

Two theoretical frameworks were used to guide the thematic analysis of participant responses. All questions that focussed on understanding the participant’s perception of the increased burden of IHD in rural areas and how this translated into actions relevant to health policy were analysed using the lens of the *Conceptual Framework for Understanding Rural and Remote Health* [23]. This framework was chosen as it explicitly focuses on understanding rural health, and the issues experienced by rural populations in order to achieve optimal health status in the Australian context. It is comprised of six concepts summarised in Table 2 [21].

**Table 2:** Summary of the six categories of the conceptual framework for understanding rural and remote health (Bourke et al., 2012).

Framework category	Summary of rural concept
**Rural locale**	Acknowledges the complex interplay between social relations, social capital, culture and country on influencing health outcomes within a geographical rural area. For example, strong social norms within a rural community regularly exist and can ultimately influence the health of that community.
**Geographical isolation**	Refers to spatial/physical distance, such as the distance of a rural locale to services.
**Health responses in the rural locale**	Includes the actions of health services/ programs in response to the rural locale.
**Broader health systems**	Broader health systems refers to how rural health systems are influenced by the actions of funding bodies, health policy, media coverage, non-government organisations.
**Broader Social structures**	Multiple structures at societal level interplay with the rural locale, geographical isolation and health systems to contribute to the current situation in rural health (such as political pressures).
**Power**	Power is both an enabler and inhibitor to change and progression within rural health, and it interacts at all levels of the framework, from the rural locale, to broader social structures influencing the health outcomes of rural Australians.

This framework used to define the context of rurality Australia and its influence over the use of scientific evidence in health policy. *The Conceptual framework for Context-based Evidence-Based Decision-Making* [15] was used to define the different stages pertaining to the use of scientific evidence in health policy decisions. This framework specifically acknowledges the rurality of a population as an external contextual influence on the use of evidence in decisions around health policy, and is especially relevant to this research, and is summarised in Table 3 below.

**Table 3:** Summary of the three stages of evidence use as outlined by conceptual framework for context-based evidence-based-policy decision making (Dobrow et al., 2004).

Stage of evidence use in decision making	Summary of concept
**Introduction**	Issues relating to the identification, accessibility, availability and rate of transmission of evidence.
**Interpretation**	This stage describes activities relating to the synthesis, evaluation and assessment of generalisability/ appropriateness of the use of evidence to the policy decision/action
**Application**	Final step in evidence based policy making where evidence is directly used to justify or determine a policy action/design

**Fig 1:**
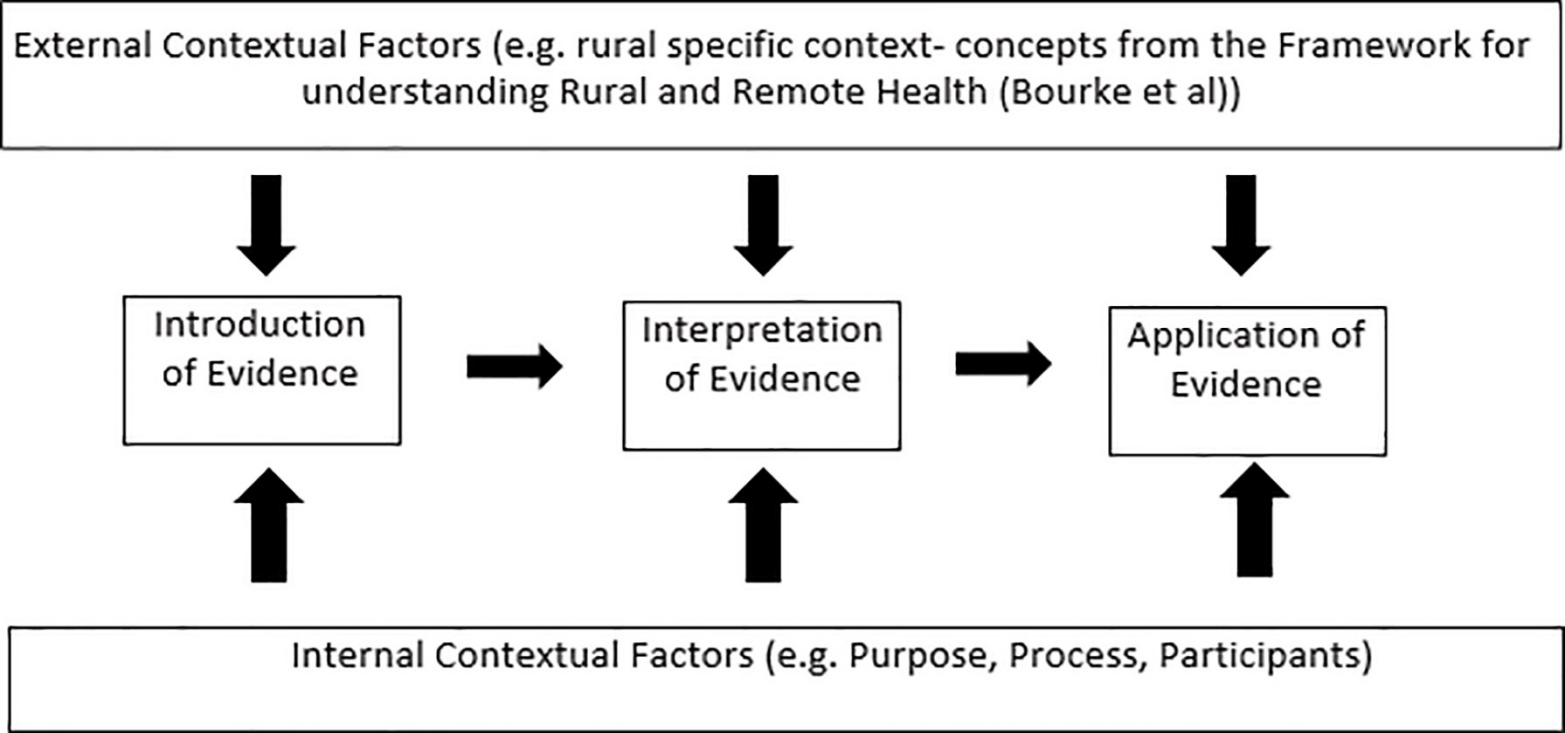
Applying the conceptual framework for understanding rural and remote health (Bourke et al., 2012) to the framework for context-based evidence-based decision making (Dobrow et al., 2004).

To ensure the specific rural context could be analysed in terms of its influence over the use of scientific evidence in health policy, these two frameworks were combined to create 18 possible themes that could be analysed within the data, using a deductive analysis approach[27]. Fig 1 shows how these two frameworks were combined when considering the use of evidence in health policy in rural areas.

NVivo software version 11 (QSR international), was used to generate, organise and analyse themes that emerged from the transcript data. Firstly transcripts were read in full by the researcher using an open coding process. Axial coding[29] was then used to analyse each theme and explore how each theme may be related. Finally, selective coding, with application of the two frameworks, was employed to generate a story from the data. LA coded the data in consultation with co-author SA and the two frameworks. Perspectives within the outlined themes were compared between participants at different levels of government.

No differences were observed in the participant responses between those working at either state or national level and so they are referred to as collectively ‘higher level’ (HL) government.

## Results

The summary of findings from each theme derived from the frameworks is summarised in Table 4. The themes of the ‘Rural Locale’, ‘Broader Health Systems’ and ‘Power’ were most prominent in the results and are discussed in additional detail below.

**Table 4:** Summary of the theoretical thematic analysis to show the influence of the Australian rural context on the use of the evidence in the policy making process for the prevention of IHD.

Stages of evidence use (Dobrow et al,2004)	Conceptual Framework For Understanding issues in Rural and Remote Health (Bourke et al,2012)					
	Rural locale	Geographical isolation	Health responses	Broader Health systems	Broader social systems	Power
**Stage 1: Introduction to the evidence (issues related to the identification, access and availability of the scientific evidence)**	Current culture within LG is to focus on SDOH as a whole, so not looking at IHD, or accessing evidence. HL:—plenty of access to the evidence at this level, however they acknowledge how the influence of the rural locale can mean that there is reduced pressure for the formulation of evidence-based policy for IHD prevention.	LG increased distance from metro areas means less resources and skilled staff in terms of being able to access high quality evidence. As distance increases- there is less access to scientifically skilled staff. HL: not really influenced as more resources and often based in bigger centres with more staffing (e.g. Canberra), but agree that geography has direct effect on LG’s ability to access evidence.	LG: Health services viewed as having the main role in accessing scientific evidence to inform specific policy on health conditions, not the LG. Also viewed as more likely to have adequate staff and resources to do so. HL: LG should play an active role in prevention of diseases and include disease specific policy.	LG: Inadequate funding from the higher levels of government and funding bodies mean there are not enough resources to be able to afford access to scientific data bases and adequately trained staff. HL: enough resources to access the evidence/ consult with experts/ NGO’s etc. at national level.	LG: Overall lower education levels in a rural community, means people in the community may be less concerned with diseases like heart disease, and therefore staff working at LG may feel less pressure to be sourcing high quality scientific evidence to justify actions. HL: Higher education levels of personnel working at HL mean evidence is more easily accessible.	LG: predictable voting patterns in rural areas mean less political pressure and therefore access to the evidence. Communities have power when they use community consultation to create pressure of prioritising issues, not always in favour of IHD related action. HL: power of NGO’s and highly educated policy advocates who have adequate resources and access to scientific evidence for policy creates power at higher levels to advocate for changes to improve IHD outcomes in rural areas.
**Stage 2: Interpretation of the evidence (includes the synthesis, evaluation and assessment of the generalisability of the evidence to policy making/decisions)**	LG: Culture/social norms within the community don’t always align with the evidence, therefore evidence is interpreted as less relevant by policy makers who interact with the rural locale. HL: Culture within advocacy team can affect if the evidence is interpreted as relevant to rural areas or not. Agreeance with LG that if the local community see’s evidence as irrelevant, then it’s acceptable to disregard its use.	LG: Research based in metro areas not interpreted to be appropriate as doesn’t account for the impact of physical spatial differences. There is the view that data would need to be small area level from rural communities to be applicable to policy. HL: Conflicted views, some agree with LG view in that geography means less likely to have scientific staff, but other participants feel that data can be generalised at larger levels than the current perception of needing community specific data.	LG: Collaboration with local health services are more likely, therefore can change views of scientific evidence and applicability to policy.	LG: As above, access is affected by inadequate funding, which means there is not sufficient time for policy maker’s to be able to analyse and make assessments about the evidence and its relevance to policy. Policy makers also reported low confidence in interpreting scientific evidence accurately due to a lack of time and professional development funds. HL: As above, there appears to be sufficient resources to encourage adequate access and interpretation of the evidence, but more focus on cancer in terms of funding for specific diseases.	As above, access to the evidence, and education levels in rural communities interact with the demand adequate resources and pressure to analyse it’s suitability for policy or action around IHD. HL: Current interpretations of evidence suggest that there is no evidence-based solution to overcoming SDOH related issues in the rural context and how this could improve the burden of IHD.	LG: Councillors views of an issue, such as heart disease can dramatically affect how the interpretation of scientific evidence and therefore they control the power of the influence of evidence over policy. HL: As above, higher level decision makers such as state or federal politicians, like councillors, have the power to interpret even the most rigorous evidence on IHD in rural areas as invalid in the policy space. Rural communities have less power due to smaller population numbers and conservative voting patterns.
**Stage 3: Application of the evidence (the evidence is applied and used to justify a policy related action/decision)**	LG: Scientific evidence is rarely used to justify policy or programs and especially in terms of the prevention of IHD. HL: rural locale not mentioned as specifically changing to this stage, depending on influence of the rural locale at stage 1 and 2.	Application is limited due to barriers at access and interpretation stages that are influenced by geographical isolation. HL: same as LG, application of evidence also inhibited by view that geography is a barrier in itself to determining solutions to rurality and emergency IHD treatment	LG: As above, interpretation and application are closely linked.	LG: due to broader health systems, many funding related barriers mean application of evidence is challenging.	LG and HL: Evidence is applied but viewed as ‘the norm’ and doesn’t have much persuasive pull as “everyone has evidence” in the political realm for a variety of advocacy or policy proposals- power has a stronger influence.	LG: As above, if decision maker’s managers in LG’s interpret the evidence to be inapplicable to their community, or if the issue of heart disease isn’t viewed as a priority, evidence is disregarded in terms of policy. The community could have power over this as they too may not believe in the evidence. HL: Evidence is applied in advocacy but not necessarily eventuating to policy action for rural areas due to differing political pressures.

Abbreviations: IHD = cardiovascular disease, HL = higher level, LG = local government, NGOs = non-government organisations, SDOH = social determinants of health

### The rural locale

The context of the ‘rural locale’ [21] had implications for all stages of evidence use within the policy process in rural areas.

#### Stage 1: Introduction of evidence

The participants at LG level described how the rural locale may play a role in likelihood of them identifying and accessing the scientific evidence on IHD in rural areas during the policy writing process. For example, in one rural area, the community felt that the high burden of IHD was a prominent issue which then led to action and evidence informed policy within the community.

“We’ve had an initiative that’s been going for 10 years which is a preventative health collaboration initiative, which it was called (omitted) … It’s called that because it was about heart disease.”—Local Government Community Services Manager and health policy writer

Other LG participants working in different rural settings thought that focussing on IHD in their policies was not useful as this was not perceived as an issue within the social norm of their community. As a result of the conditions within these settings there was unlikely to be IHD specific policy, and therefore policy makers would be even less likely to be accessing the relevant scientific evidence on the issue.

“*We know that what we’re doing is to eventually prevent chronic disease*, *but focusing on the chronic disease itself*, *we haven’t found it’s particularly effective when it comes to communicating with the community*.*”* -*Social planner responsible for public health and health policy*

One LG participant highlighted how within their rural locale, using scientific evidence in their policy work was atypical.

“Probably the short answer is no. I probably don’t chase any of that sort of stuff (scientific intervention studies).”- Environmental Officer responsible for public health and health policy

At the HL, the ‘rural locale’ was also acknowledged as having influence over whether or not there would be pressure on the LG to develop evidence based policy and strategies for reducing IHD. The following quote is a reflection of how the perceptions of the community can influence risk behaviours, or make them socially acceptable and therefore reducing pressure on LG to form policy related to preventable diseases such as IHD.

“*So*, *each of those towns have its own cultural identity and people in those towns*, *you know*, *to a greater or lesser extent operate within a unique cultural environment*, *if you like*. *So*, *an environment where it's*, *you know*, *if you’re not smoking what's wrong with you*?*”–Rurally-based National Policy Advisor*

#### Stage 2: Interpretation

The social norms within the rural locale of LG participants had a direct effect on how the scientific evidence is interpreted and considered as relevant to health policy action. LG participants working within the rural locale are influenced by the social norms and structures within their rural area, as they interact with their unique surroundings. If the evidence doesn’t feel right, or fit in with the perceived culture then it was less likely to be interpreted as valuable and required for the progression of policy work around IHD prevention.

“*If you present me with something and I don’t think that that makes intuitive sense*, *I’m going to be sceptical about using it as—as evidence*, *which is*, *I know*, *completely unscientific……*.*Then you see*, *you’ve always got to be a little bit careful about studies and—and research”–Director of Community Services responsible for designing public health and health policy*.

The influence of the rural locale is evident in the view that if scientific studies are not generated from within the rural community then they are interpreted as less relevant than the local community stories when justifying policy actions around preventable conditions such as IHD.

“*It’s international*, *or it’s*, *you know*, *it’s urban or something like that… you think well*, *it does really have to be relevant to the area*. *And as I said*, *communities are all different*, *and even though a lot of the health issues and cardiovascular issues in (name) Shire are quite similar to (neighbouring shire)*, *you know*, *it’s completely different local government area*. *So I would be needing to know that actually that scientific study is going to be relevant for our community*.*”–Director of Community Services with experience in multiple rural local governments in policy formulation*

In contrast, a senior government data analyst felt there was not a need for small area level data to support policy action in rural areas. The participant felt that despite spatial heterogeneity across rural communities, the evidence on disease burden in rural Australia would be generalizable to most of these communities and therefore should be applied to policy.

“Most of the time that you can use stuff at a much higher level than people want; the exception to that is if you want to investigate where an area has put in place a particular practise.”-Senior Government Data Analyst

The next quote captures the influence of a rural locale over the interpretation of scientific data. Anecdotal stories from the community are seen as ‘real data’ that is relevant to them, when compared to scientific studies:

“I would probably say case studies are a good one…because they’re real life studies, usually. It’s real data. It focuses on a specific… like, a lot of time, a case study might focus on a specific group of people, or a specific person. It’s a case study about their experiences and the outcomes, and those sort of things”- Director of Community Services responsible for health policy formulation

HL participants agreed that the rural context has strong influence over the interpretation of the evidence within policy teams. There was agreement with the LG that evidence had to be palatable to the rural locale involved, and that people working in rural environments with access to context-specific evidence were in a position to develop more innovative and potentially effective responses.

“I think that often, some of the best new pilots, and looking at things to do differently come from local people who’ve put the effort in to get that evidence–as opposed to ideas that come out of Melbourne from the departments.”–State Member of Parliament (rural electorate)

#### Stage 3: Application

Due to barriers related to the rural locale, such as the lack of pressure to access and use the evidence in justifying policy around IHD prevention, application of the scientific evidence was viewed as rare, and in some cases had not been used at all at the local level.

“It hasn’t happened before” -Social Planner and Health Policy Writer

At the higher level, one participant suggested that the current system within LG should change to align with the evidence around IHD and despite the effect of the rural locale, be applying evidenced-based policy and taking direct action that is less general and more disease specific.

“Well there is a kind of wishy-washy school of health promotion that thinks we shouldn't mention diseases. It seems to me a bizarre notion.”–Senior National Policy Advisor

One participant felt that the rural locale held back communities from being seen as a political priority because rural populations were generally predictable voters; which relates back to culture, social norms and social rules within rural communities. Without political focus on the issue there would be less motivation to generate and apply the scientific evidence to the policy, if no policy was likely to form in the first place.

“I don't think rural health is a—is an electoral agenda at all. And—and the—and the raw calculus of it is that you, you know, most rural areas vote conservatively and so, again, you know, if that seat's not within—if you're polling and it tells you that the swing—the swing is on or it's too tight, then do what you have to hold it, but, geez, don't—don't go wasting time and money and resources in a seat that's already safe.”-National Senior Policy Advisor

On a similar theme to the quote above, another participant acknowledged how the characteristics of rural communities also did not encourage evidence based policy generation in these areas, due to little direction or knowledge of adequate evidence based solutions:

*“We find that people in rural areas tend to have lower incomes*, *have lower levels of educational attainment…… and no one's really nailed it in terms of how you can sort of level the playing field for rural and remote populations versus urban populations*.*”*- *Senior National Policy Advisor*

### Broader health systems

The influence of the broader health system was evident in the participants’ responses about the use of evidence in policy relating to IHD prevention in rural areas, predominantly in relation to a lack of funding and resources to generate evidence-based policy.

#### Stage 1: Introduction

The ability of policy makers working at LG level to access the scientific evidence in rural areas was limited by a lack of resources and funding specific to reduced/ or no access to scientific databases and therefore the scientific evidence. This makes the development of rural health policy that incorporates scientific evidence specific to rural communities difficult to achieve.

*“Studies are obviously like pay for—like you need to pay to access the article and stuff like that…*.*which is a pretty massive*, *um*, *barrier for an organisation that has really low resourcing levels*.*”* -*Social Planner and Health Policy writer*.

At the HL, the opposite situation was observed as funding for such staff in advocacy and advisory roles did not appear to be an issue as these participants worked in larger organisations with higher levels of resourcing. It was obvious to participants at the HL that the rural LG policy makers were under-funded and under-resourced in terms of being able to access evidence, and one HL policy maker agreed:

“*if the political process is what allocates resources for the management*, *care and prevention of heart disease in rural areas*, *then you work backwards and go*, *"Well*, *why isn't there*? *Why isn't there more funding*, *more resources*, *you know*, *um*, *you know*, *a better workforce*?*"- National Senior Policy Advisor*

#### Stage 2: Interpretation

As well as having limited or no access to scientific literature, there were also no identified funding mechanisms for staff to interpret and analyse the evidence related to IHD prevention in rural areas. Due to the lack of funding around training and support, LG participants felt low confidence in interpreting the evidence accurately. When asked if they felt confident interpreting the scientific evidence one participant said:

“I could be left red faced if somebody put a very scientific study in front of me”- Environmental Planner and Health Policy Writer

Again, at the HL there did not appear to be barriers to interpreting the evidence, especially in regards to funding and resources to develop advocacy or policies that incorporated scientific evidence.

#### Stage 3: Application

Application of the evidence was reduced by the influence of broader health systems at LG, however this was not a prominent issue at HL. There was potential for broader health systems to contribute to improving access to evidence on IHD in rural areas for LG participants. One participant suggested that there was a lack of data sharing and an improved system to encourage better data keeping and streamlined data collection between local governments and health services could assist with the generation of rural-specific scientific evidence on issues such as IHD. Generation of such evidence would be viewed as more applicable to their work as it would account for heterogeneity between rural areas.

*“We’ve just got an extraordinary amount of information*. *The problem is*, *is that I don't know what the local hospital has got*. *They don't know what the shire has got*. *They don't know what the community health service has*?*”***–***Community Services Director and Health Policy Writer*

### Power

Examples of power influencing each stage of the evidence use process were evident in this study, and are summarised together due to the closeness of the rural context interactions at each stage.

At LG, rural communities demonstrate power through participating in the community consultation processes. Policy makers at the LG felt that community consultation was more of a priority than using scientific evidence, as the issue had to be marketable to their local community. Views within the community collectively have the power to inhibit the use of scientific evidence, and especially around accessing evidence on the issue of preventing IHD. The following quote captures the perspectives of the participant in feeling that the evidence is not necessarily worth accessing if the community decides that the issue is not one of concern.

*“How do you market the issue*? *If you're saying we have a death rate higher from heart disease than most other townships*, *people are going to say*, *"Well*, *I'm not old yet so it doesn't impact on me*.*"…*..*But if we're talking about obesity …*..*You don't see a heart ready to have a heart attack in the street*.*”*-*Local Government Health policy writer*

Participants at LG did however feel they could have the power to take actions and develop evidence-based policy to reduce the burden of IHD in rural areas.

“I think local government has the opportunity to have a big impact over the longer term.”- Health policy developer

At the HL, politicians were viewed as having significant power over the interpretation and application of evidence in policy in rural areas. Participants shared the view that regularly, despite the best available evidence and advocacy around heart disease, rural communities missed out due to the influence of power in political circles. This participant sums up how power in politics has a big influence over how the evidence is accessed, interpreted and applied, and in this quote implied that evidence has little power at all.

“Academics and political scientists may as well be talking about the lifecycles of grasshoppers for all the influence it has in a prime minister's office.”-Senior National Policy Advisor

There was discussion around a lack of power for politicians who do have a personal background in rural health, and despite having extensive knowledge of rural health issues and barriers to reducing the IHD burden, still had very little power to create change within the current political environment.

“I just don't think she’s (Federal Member of Parliament) ever been given any resources to do anything. Nor is it filling the mail bags as they say. People are not filling MP's mailbags saying, "Look, we've got a higher rate of this that and the next thing in rural areas and we ought to do something about it…” At the higher policy level there's at best inertia and at worst vested interests at work which are operating against the things which would have—would be bringing benefit to rural areas in terms of reducing the heart attack rates, i.e. preventative activities”- Senior National Policy Advisor

Power in rural areas, in a political sense, was also perceived to be reducing over time as metropolitan areas were expanding, creating stronger centres of power in capital cities. This therefore would reduce the likelihood of a government focus on rural health and the development of evidence-based policy to reduce IHD in rural Australia.

“We lost one seat–one country seat–in country Victoria. So as a result, we’ve got one fewer, one less voice in parliament that’s advocating for investment in rural health, for example.”- State Member of Parliament (rural electorate)

## Discussion

### Main findings

This research set out to understand the perceptions of rural policy makers on the use of evidence in their efforts to set policy to reduce heart disease in rural and remote populations. The rural Australian context appears to be a key variable reducing the likelihood of the development of evidence-based policy to reduce the high burden of IHD experienced by rural communities. The data collected here suggest that the lack of resources available to rural policy makers prevent meaningful use of scientific evidence in policy making. Specifically, lack of access to data relevant to their community, social norms within the rural locale, limited funding and lack of skilled staff in rural settings have inhibited their ability to apply scientific evidence to the policy making process.

At a national level the resources to support rural and remote health allow for the rapid creation of evidence briefs and background summaries to support policy making at national and state level. However, political processes and the perceived lack of power of rural populations in parliament mean that focus on the specific needs of rural populations the adoption of scientific evidence-based policy is limited at the federal level.

This study used a purposive sampling approach to identify key people in rural policy making and seek their perspectives on the role of evidence in rural policy making. We applied a snowball sampling approach[27] whereby those in a role or with experience relevant to the study question were invited to participate. A significant strength of this study is that this recruitment process resulted in a very informed sample and a second strength is that all key informants approached agreed to participate.

This is the first study to interview rural policy makers with a heart disease focus at all three levels of government and this has demonstrated a fundamental difference in perspective and approach between the State/ Federal level of government and the local rural level. The application of the rural health framework [21] in conjunction with the context-based evidence-based decision making framework [15] is also novel and this has helped to explicate the key aspects of evidence use in rural policy notably the role of context, resource and skill mix.

A potential limitation of this study is that the sampling approach relied on the potential key informant being accessible on the internet in the first instance, and recall of their colleagues skill set or experience in the case of the snowball sampling. The rural informants to this study were Victorian based and this may limit the generalisability of these findings to others states of Australia. Indeed, the policy context would be different in other states; of the eight Australian states and territories only New South Wales is similarly mandated as Victoria to create health and wellbeing plans at a local government level. Replication of this work in other states and territories might expect to find far fewer people working in rural IHD health policy with perhaps even fewer resources at their command.

Application of the conceptual framework for understanding rural health has demonstrated how the rural context affects the policy making process. A possible reason that current health policy is not meeting the needs of rural Australians emerged in the perspectives of higher level government participants. The majority of respondents suggested that reduced political power in rural areas makes effective and specific rural health policy a low political priority. Participants perceived that political pressure is lacking from rural Australia and therefore politics is becoming increasingly ‘metro-centric’ in part due to expanding urban populations, which is also echoed in the Australian literature [10,21]. Based on the participants’ views, persistent inequities in the burden of heart disease in rural Australia could be considered through the theoretical lens of the ‘political economy theory’ [30,31]. This presents the view that health inequalities in affluent societies (such as Australia) may be an outcome of ‘the social and politically mediated exclusion from material resources’, as described by the theme ‘broader social structures’ [30]. For example, the allocation of material resources (adequate training for staff to produce appropriate evidence based health policy) to rural populations is a major implication for the potential to reduce IHD in rural Australia, or preventable diseases as whole.

Rural Australia is no exception to observations in the current international literature that evidence is only a small influence on the decision making processes, with many other factors having more dominant influence, such as political pressures [15,18,20,32]. A systematic review of the use of scientific evidence in international health policy also found similar results to those described here including multiple barriers to the use of scientific evidence such as perceptions of the relevance of the evidence and decision making cultures [33].

The most recent rural specific policy in Australia is the National Framework for Rural and Remote Health (2011 [9], which set a vision that rural and metropolitan Australians will achieve equal health status. The framework document acknowledges there is less public funding allocated to rural health care resources when compared to metropolitan areas, despite rural health services being more expensive to operate [9]. This has left the rural health sector largely under-resourced, creating a larger barrier to improving health outcomes for rural populations [9]. As an example of the difficulty, the Victorian Municipal Health and Wellbeing Plan (MHWP) represents the legislative requirement under the Public Health and Wellbeing Act (2008) at local government level in Victoria, Australia [28]. Despite the well-known funding and health outcome inequities, rural or regional communities are not considered as a community of need, nor identified directly, in this legislation.

### Implications and future questions

This study found clear evidence that the quality and specificity of the data available to the rural health services was a barrier to the use of policy. Whether real or perceived, the lack of applicable local and rural data is inhibiting the use of evidence of evidence in policy making. To overcome this there is a need for routine, high quality local health data and subsequent analyses which are sensitive to the needs of the local community and collected with the goal of evidence informed policy in mind.

We observed a significant gap in the use of evidence in rural health policy making which is supported by research with similar findings in other fields. For example a study by Vujcich et al [20] that investigated the use of evidence in policy decisions around reducing Aboriginal tobacco smoking rates also found that there were many other factors that influenced decisions around policy, and that high quality scientific evidence was not always perceived to be accessible to policy makers.

Future health policy research needs to be sensitive to the nuance of the rural location and understand the role of context in the making and implementation of policy for IHD prevention. Further work is needed to understand why evidence is not explicitly incorporated into policy, through the lens of ‘rural locale’ provided by the conceptual framework for understanding rural and remote health. Further research into the views of rural communities on the need for evidence-based rural health policy would also offer rich insights into the policy process, as the participants in this study cited community stores and perceptions to be more powerful than scientific evidence when influencing policy decisions at the local level.

Reform of the Public Health and Wellbeing Act (2008) to include considerations for rurality, could indeed be beneficial in assisting local rural governments to improve the poorer health outcomes experienced by rural communities, and would assist with the recognition that there is a unique rural distinction in policy making, as acknowledged within the conceptual framework for context based evidence-based decision making (15).

## Conclusions

Despite large advances in heart disease prevention globally this remains a key area of inequality between urban and rural dwelling Australians. The use of scientific evidence in health policy is influenced by multiple factors which is recognised on an international scale, and our findings show that the rural context leads to conditions which constrain the ability of the Australian government to focus on these inequalities and subsequently to apply evidence to their efforts in prevention. If these contextual inequalities are not addressed, the inequities in morbidity and mortality will persist for future rural-dwelling communities.

## Supporting information

S1 File Outline of interview questions. (DOCX)
