# Supplementary material for: Policy makers’ perceptions of the high burden of heart disease in rural Australia: Implications for the implementation of evidence-based rural health policy
Source: PLoS One. 2019 Apr 16;14(4):e0215358. doi: 10.1371/journal.pone.0215358 (PMC6467412; doi:10.1371/journal.pone.0215358)
Supplement: S1 File — (DOCX) [file pone.0215358.s001.docx]

*Interview schedule:*

**These questions were used as guide only and interviews were semi-structured to allow for emergence in a variety of themes and subject discourse.**

**Outline of introduction to be given by interviewer:**

The purpose of this research is to gain understanding into your opinions and experiences with rural health policy especially in terms of reducing ischaemic heart disease in rural areas of Australia. This may help us to understand ways to improve rural health policy and prevention efforts for the future.

1. Tell me about your current/previous roles and how this could relate to prevention of heart disease in rural areas?
2. How does policy fit into your current/previous role?
3. What do you view as the major health concerns for the rural population currently?
4. Ischaemic Heart disease rates are known to be higher outside of major cities, in your experience, how is this increased burden viewed? Is it a prominent concern for policy makers or people in your area of work?
5. Can you give examples of past policies, or policy changes that are likely to influence heart disease in rural areas?
6. Are there any health policies in this area that you would like to see happen that have not been implemented or adopted?
7. Experiences with using the scientific evidence to design policy/prevention:
8. What do you see as ‘evidence’ that you might use as information to make a decision related to policy/prevention?
9. Do you feel that priority is placed on reviewing the scientific evidence before making decisions on rural health policy?
10. In your experience, to what extent is the scientific evidence consulted when making decisions about policy/prevention in rural areas? (If participant is unsure of what is meant by ‘scientific evidence’: Definition of what is meant by ‘scientific evidence’: Published research studies, national data sources (e.g. mortality data), and government reports such as those provided by the Australian Bureau of Statistics and Australian Institute of Health and Welfare.)
11. What are the barriers to applying the scientific evidence when designing policy to reduce heart disease in rural areas, if any?
12. Do you feel that you have access to the evidence you need to understand rural health issues? What about specifically for heart disease?
13. How confident do you feel in using and interpreting the scientific evidence on health inequalities in rural Australians, when making decisions or acting on policy?
14. Why/why not? Is there room for improvement if the answer is yes?
15. Do you feel you could benefit from more support in understanding the scientific evidence on this issue?( In terms of support, this could be access to expert researchers in the field, or further education in interpreting scientific papers, data etc.).
16. I have here a number of examples of types of evidence from different sources. I would like you to have a look at these and think about which types of evidence you see as the most powerful. Please arrange the cards in order from most to least [significant], and explain as you go why you have ranked different options highly or poorly.
17. If you could choose one risk factor that you think would make the biggest impact on reducing heart disease in rural areas, which one would it be? How much would you expect to be able to reduce it by through policy changes?
